# Immune Checkpoint Blockade Augments Changes Within Oncolytic Virus-induced Cancer MHC-I Peptidome, Creating Novel Antitumor CD8 T Cell Reactivities

**DOI:** 10.1016/j.mcpro.2021.100182

**Published:** 2021-12-16

**Authors:** Youra Kim, Prathyusha Konda, J. Patrick Murphy, Joao A. Paulo, Steven P. Gygi, Shashi Gujar

**Affiliations:** 1 Department of Pathology, Dalhousie University, Halifax, Nova Scotia, Canada; 2 Department of Microbiology and Immunology, Dalhousie University, Halifax, Nova Scotia, Canada; 3 Department of Biology, University of Prince Edward Island, Charlottetown, Prince Edward Island, Canada; 4 Department of Cell Biology, Harvard Medical School, Boston, Massachusetts, USA; 5 Department of Biology, Dalhousie University, Halifax, Nova Scotia, Canada

**Keywords:** Oncolytic reovirus, immune checkpoint blockade, cancer immunotherapy, MHC-I peptidome, antitumor immunity, DEMHCP, differentially expressed MHC-I-associated peptide, ICB, immune checkpoint blockade, IFNγ, interferon gamma, IP, immunoprecipitation, MHC, major histocompatibility complex, OV, oncolytic virus, PD-1, programmed cell death protein 1, PDL-1, programmed death-ligand 1, TB, tumor-bearing, TME, tumor microenvironment, TMT, tandem mass tag

## Abstract

The combination cancer immunotherapies with oncolytic virus (OV) and immune checkpoint blockade (ICB) reinstate otherwise dysfunctional antitumor CD8 T cell responses. One major mechanism that aids such reinstatement of antitumor CD8 T cells involves the availability of new class I major histocompatibility complex (MHC-I)-bound tumor epitopes following therapeutic intervention. Thus, therapy-induced changes within the MHC-I peptidome hold the key to understanding the clinical implications for therapy-reinstated CD8 T cell responses. Here, using mass spectrometry–based immuno-affinity methods and tumor-bearing animals treated with OV and ICB (alone or in combination), we captured the therapy-induced alterations within the tumor MHC-I peptidome, which were then tested for their CD8 T cell response-stimulating activity. We found that the oncolytic reovirus monotherapy drives up- as well as downexpression of tumor MHC-I peptides in a cancer type and oncolysis susceptibility dependent manner. Interestingly, the combination of reovirus + ICB results in higher numbers of differentially expressed MHC-I-associated peptides (DEMHCPs) relative to either monotherapies. Most importantly, OV+ICB-driven DEMHCPs contain biologically active epitopes that stimulate interferon-gamma responses in cognate CD8 T cells, which may mediate clinically desired antitumor attack and cancer immunoediting. These findings highlight that the therapy-induced changes to the MHC-I peptidome contribute toward the reinstated antitumor CD8 T cell attack established following OV + ICB combination cancer immunotherapy.

## Highlights

• Reovirus-mediated change to MHC-I peptidome depends on tumor type and oncolysis.
• Addition of ICB augments changes to MHC-I peptidome of reovirus-resistant tumors.
• REO+ICB therapy-modulated MHC-I peptides stimulate CD8 T cell responses.
• Not only upregulated but also downregulated MHC-I peptides can stimulate CD8 T cells.

## Graphical Abstract

**Figure:**
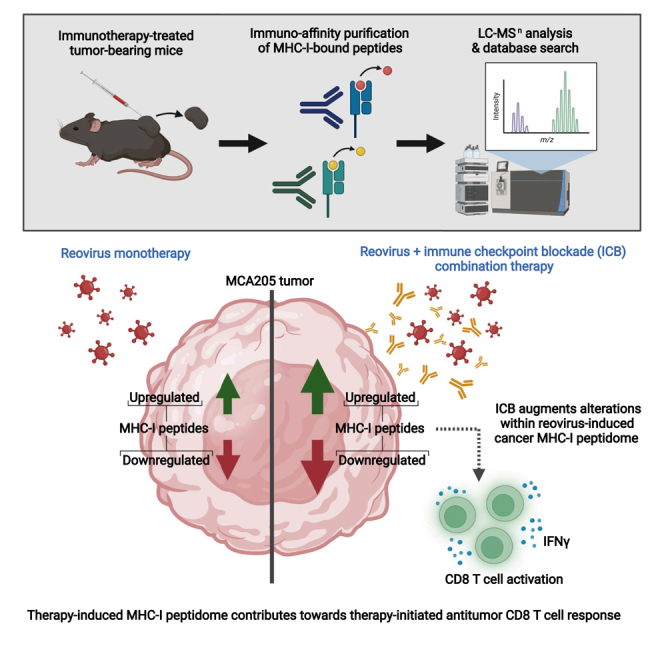

## In Brief

Immune checkpoint blockade augments changes within oncolytic virus-induced cancer MHC-I peptidome and contributes toward the therapy-induced novel antitumor CD8 T cell reactivities.

Immunotherapies aim to (re)educate the immune system to recognize and eliminate cancer cells, and unlike conventional anticancer therapies, the resulting antitumor immune response can provide a highly specific and long-lasting protection (1, 2). These cancer immunotherapy approaches often focus on overturning the immunosuppression mediated through diverse immune evasion mechanisms within the tumor microenvironment (TME). In particular, cancer immunotherapy approaches based on the blockade of inhibitory immune checkpoints, such as PD-1, CTLA-4, and PDL-1, have shown promise in clinical settings and are being recognized for their capacity to reinstate antigen-specific CD8 T cell attack on cancers. Such therapy-induced antitumor CD8 T cell response is shaped by a spectrum of tumor antigens (*i.e.*, it is polyclonal), can act on existing cancer cells, and maintain antigen-specific memory response against tumor challenge or relapse (2, 3, 4, 5). Thus, it is not surprising that “hot” tumors—those with a higher density of tumor-infiltrating cytotoxic CD8 T cells—respond better to immune checkpoint blockade (ICB) and correlate with better clinical outcomes as compared with “cold” tumors, which show low or no immune cell infiltration in the TME (2, 3, 6, 7). Based on these observations, strategies that can make tumors “hot” and prime them for ICB therapies are being pursued in an attempt to design broadly applicable and effective cancer immunotherapies.

One way to make tumors “hot” ahead of ICB therapies involves the use of oncolytic viruses (OVs), which were originally discovered for their capacity to preferentially infect and kill cancerous cells without causing similar effects on normal cells. In the last decade, it has become clear that in addition to their direct tumoricidal effects, OVs overturn a myriad of tumor-associated immune evasion mechanisms and promote the induction of potent antitumor immune responses (8, 9, 10, 11). For instance, the type I interferon-driven response to viral infection restores the expression of proteins involved in antigen processing and presentation in various cancer cells (12, 13, 14). OVs also support the recruitment and activation of CD8 tumor-infiltrating lymphocytes, as well as other immune cells, *via* a localized release of cytokines in the TME (15, 16, 17). This tumor immune infiltration-driving action of OVs makes them a suitable candidate for making tumors “hot” and supports their use in combination with ICBs. Interestingly, the biological activities of OV monotherapy-induced antitumor CD8 T cell responses are dampened *via* the actions of immune checkpoints such as PD-1 and require rescuing *via* ICB to sustain their antitumor functions. Thus, during OV + ICB combination therapy, OVs and ICBs overcome the limitation faced by each monotherapy: OVs make tumors “hot” and suitable for ICBs, and ICBs potentiate OV-induced CD8 T cell responses (4, 5, 18). To this end, previous studies have shown the enhanced efficacy of OVs through (a)synchronous administrations of ICBs (16, 19, 20, 21, 22, 23, 24), and OVs have emerged as a strategically complementary partner for ICB therapies (25, 26).

CD8 T cells are the main mediators of OV + ICB combination therapy (16, 20, 21, 23, 24). The antigenic targets of CD8 T cells are complexes of peptides associated with class I major histocompatibility complex (MHC-I) molecules found on the surface of all nucleated cells (27, 28). These MHC-I-bound peptides are derived from proteolysis of intracellular proteins, where those that originate from normal tissue are nonimmunogenic while ones from viral proteins or abnormal tissue (mutated or overexpressed tumor-associated antigens) are immunogenic (27, 28). As such, the repertoire of peptides presented by MHC-I molecules, termed the MHC-I peptidome, reflects the health state of a cell. The MHC-I peptidome can be analyzed by a mass spectrometry (MS)-based approach using immunoprecipitation (IP)-purified peptide-MHC-I complexes (29, 30, 31). Many cancer types, however, have a defective antigen processing and presentation machinery, thereby complicating the elucidation of the tumor MHC-I peptidome landscape (32). We have recently shown that oncolytic reovirus can correct tumor-associated antigen presentation defects and promote the expression of MHC-I peptides on tumors that can induce new antitumor CD8 T cell responses. Currently, whether OV+ICB combination therapy affects the tumor MHC-I peptidome and subsequently shapes the repertoire of immunogenic antitumor CD8 T cells remain poorly understood.

The current study used MHC-I IP and LC-MS/MS with label-free or tandem mass tag (TMT)-based multiplexed quantitation to analyze the tumor MHC-I peptidome following OV + ICB combination treatment. We found that oncolytic reovirus-mediated changes to the MHC-I peptidome *in vivo* are cancer-type-specific, where differentially expressed MHC-I-associated peptides (DEMHCPs) displayed quantitative and qualitative variance in a tumor-model-dependent manner. The addition of ICB to reovirus therapy showed potential therapeutic value since a greater change to the MHC-I peptidome was observed due to the combination therapy compared with either monotherapy alone. These DEMHCPs were capable of inducing antigen-specific CD8 T cell responses in reovirus + ICB-treated tumor-bearing (TB) mice, but not in nontreated TB mice. Such therapy-induced changes within the MHC-I peptidome and inherent changes in CD8 T cell activity may improve antitumor immunity and hold biological as well as therapeutic importance (33).

## Experimental Procedures

### Reovirus, Cell Line, and Reagents

Reovirus (serotype 3, Dearing strain) was cultured, amplified, and isolated using a previously established protocol (34). Mouse ovarian surface epithelial cell line (MOSE, clone ID8) was obtained from Dr Edith Lord (University of Rochester) (35), and mouse MCA205 fibrosarcoma cell line was obtained from Dr Guido Kroemer (INSERM) (36). Both cell lines were grown at 37 °C, 5% CO_2_ in DMEM containing 10% (vol/vol) fetal bovine serum, 1× sodium pyruvate, 1× nonessential amino acids, and 1× Antibiotic-Antimycotic (all obtained from Invitrogen). *InVivo*Mab anti-mouse PD-1 antibody (clone 29F.1A12) and *InVivo*Mab rat IgG2a, κ isotype control antibody (clone 2A3) were purchased from Bio X Cell. Purified anti-mouse MHC-I antibodies were produced in-house from hybridoma clones B22.249 (H2-D^b^ specific) (37) and Y3 (H2-K^b^ specific) (38). TMT10plex isobaric label reagent set plus TMT11-131C label reagent was purchased from Thermo Fisher Scientific. Peptides were purchased from JPT Peptide Technologies. The following antibodies were purchased from BioLegend: APC anti-mouse H2-K^b^ (clone AF6-88-5), functional grade purified anti-mouse CD28 (clone 37.51), FITC anti-mouse CD4 (clone RM4-5), PE anti-mouse CD3ε (clone 145-2C11), PerCP-Cy5.5 anti-mouse CD8a (clone 53-6.7), PE anti-mouse PDL-1 (clone 10F.9G2) and FITC Annexin V. FITC anti-mouse H2-D^b^ (clone 28-14-8), APC anti-mouse IFNγ (clone XMG1.2), F(ab’)2-goat anti-rat IgG (H + L) secondary antibody, 7-amino-actinomycin D (7AAD) viability staining solution, and Foxp3/transcription factor staining buffer set were from eBioscience. *InVivo*Mab anti-mouse CD16/32 (clone 2.4G2) was from Bio X Cell. Rat anti-reovirus polyclonal antibody was produced in-house. Anti-mouse IFNγ DuoSet ELISA kit was purchased from R&D Systems.

### *In Vivo* Experimental Procedures

All *in vivo* experimental procedures were approved by the University Committee on Laboratory Animals (UCLA) at Dalhousie University. Six- to eight-weeks-old female wild-type C57BL/6 mice were obtained from Charles River Laboratories. Mice were injected as follows with the frequency of treatment shown in the figure schematics: reovirus (5 × 10^8^ plaque forming unit [PFU], intratumorally [i.t.] or intraperitoneally [i.p.]), MCA205 (5 × 10^5^ cells, subcutaneously [s.c.]), anti-mouse PD-1 antibody (250 μg/mouse, i.p.), rat IgG2a, κ isotype control antibody (250 μg/mouse, i.p.).

### Antibody Staining for Flow Cytometry

MCA205 and ID8 cancer cells were infected with reovirus at multiplicity of infection (MOI) of 0, 10, 100, 1000, and 10,000 for 24 h *in vitro*. Stimulation with mouse IFNγ at 100 units/ml (U/ml) was included as a positive control. Cells were collected and stained with anti-mouse H2-D^b^, H2-K^b^, and PDL-1 antibodies for 25 min at 4 °C in flow cytometry running buffer (PBS-EDTA with 1% FBS; FACS buffer), fixed with 1% paraformaldehyde (PFA) for 15 min at room temperature (RT), and resuspended in FACS buffer prior to analysis. To measure reovirus infectivity, cells were permeabilized in 1% Triton X-100 (Sigma-Aldrich; vol/vol in PBS) after being fixed with 1% PFA, incubated with rat anti-reovirus polyclonal primary antibody (1:500), followed by goat anti-rat secondary antibody (1:500) in 0.1% Triton X-100 for 25 min at 4 °C. To measure oncolysis, cells were stained with Annexin V in Annexin V binding buffer for 5 min at RT before 7AAD was added and incubated for an additional 15 min at RT.

Flow cytometry analysis was performed on immune cells harvested from the peritoneum and spleens of animals collected independently. Cells were harvested *via* a flush of the peritoneum with PBS-EDTA (1% vol/vol), and spleens were mechanically disrupted using the end of a syringe plunger. Harvested cells were filtered through a 40 μm cell strainer, treated with red blood cell–lysing ammonium-chloride-potassium (ACK) buffer (Thermo Fisher Scientific), washed, and blocked with anti-CD16/32 antibody for 25 min at 4 °C. Cells were then stained for CD3 and CD8 T cell markers, along with PD-1 and PDL-1 for 25 min at 4 °C in FACS buffer, and then fixed with 1% PFA. For analysis of tumor-infiltrating lymphocytes, mechanically dissociated tumor tissues were processed by Ficoll-Paque density gradient centrifugation. All flow cytometry data were collected using FACS Canto II flow cytometer (BD Bioscience), and analysis was conducted using FACSDiva (BD Bioscience) and FCS Express V6 software (DeNovo Software).

### Real-time Quantitative Polymerase Chain Reaction (RT-qPCR)

RNA extractions were performed using standard TRIzol methodology as per the manufacturer’s instructions. Purified RNA was quantified and diluted to 2 μg for the synthesis of complementary DNA (cDNA) using Superscript II (Invitrogen). cDNA was amplified and quantified using the CFX96 touch RT-PCR instrument (BioRad Laboratories), and murine gene-specific primers were purchased from Invitrogen. Primers used (5′-3′) include *Gapdh* (TGGCAAAGTGGAGATTGTTG and AAGAT GGTGATGGGCTTCCC), *Cxcl10* (GTTGAGATCATTGCCACGATGAAA and CTGCTGTCCATCCATCGCA), *Ddx58* (AGACGGTTCACCGCATACAG and AAGCGTCTCCAAGGACAGTG), *Ifnb* (CCCTATGGAGATGACGGAGA and ACTTGAGGTGGTCGTCTGTC), *Il1b* (GCCCAT CCTCTGTGACTCAT and AGGCCACAGGTATTTTGTCG), and *Tlr3* (TCCTGCTGGAAAACTGGATGG and AGCCTGAAAGTGAAACTCG CT). qPCR data were collected and analyzed using the Livak and Schmittgen’s 2^−ΔΔCT^ method (39), where fold change was calculated by first normalizing the cycle threshold (C_T_) of the indicated gene against the *Gapdh* reference gene, followed by a comparison against the respective controls.

### MHC-I Peptide Isolation

Tumor samples were collected following the treatment regimens shown in the figure schematics, flash frozen, and stored at −80 °C until processing as previously described (29, 31, 40). For each treatment group, 1 g of tumor tissue was cut into small fragments and mixed with 10 ml of lysis buffer comprised of 0.25% sodium deoxycholate (Sigma-Aldrich), 0.25 mM iodoacetamide (Sigma-Aldrich), 1 mM EDTA (Thermo Fisher Scientific), 1:200 cOmplete, Mini protease Inhibitor Tablets (Roche), and 1% octyl-β-glucopyranoside (Sigma-Aldrich) in PBS. The lysates were processed with a tissue homogenizer (three 20 s intervals, on ice), shaken gently on a rotator for 30 min at 4 °C, sonicated (three 20 s intervals, on ice), and shaken again for 30 min at 4 °C. Lysates were cleared by centrifugation at 4 °C, 3300*g* for 50 min and then precleared of endogenous antibodies using Protein-A Sepharose 4B (Pro-A) beads (Invitrogen). MHC-I complexes were immunoaffinity purified from the lysates using the B22.249 and Y3 antibodies (1 mg/g of tissue each), covalently bound to Pro-A beads with dimethyl pimelimidate dihydrochloride (Sigma-Aldrich) and incubated overnight at 4 °C on a rotator. The samples were passed through Poly-Prep columns (Bio-Rad Laboratories), and the bead-bound MHC-I proteins and peptides were washed four times with 2 ml of 150 mM NaCl in 20 mM Tris-HCL, pH 8.0; four times with 2 ml of 400 mM NaCl in 20 mM Tris-HCL, pH 8.0; four times with 2 ml of 150 mM NaCl in 20 mM Tris-HCL, pH 8.0; and then two times with 2 ml of 20 mM Tris-HCL, pH 8.0 using a vacuum manifold. MHC-I molecules and their bound peptides were eluted eight times with 200 μl of 0.2% trifluoroacetic acid (TFA). The eluates were purified by solid-phase extraction (SPE) with 60 mg Oasis HLB cartridges (Waters). Peptides were eluted from SPE with 30% acetonitrile (ACN), lyophilized, and desalted using home-made Stage-tips packed with Empore C18 extraction material (Sigma-Aldrich) as previously described (41), then lyophilized.

### Mass Spectrometry Analysis of Label-free MHC-I Peptides

For label-free MHC-I peptidome analysis, lyophilized peptides were solubilized in 12 μl of 1% formic acid and analyzed by LC–MS/MS. For each antibody eluate, an aliquot of 1 μl of peptides was injected onto a 75 μm × 30 cm column (New Objective) self-packed with 4 μm, 90 Å, Proteo C18 material (Phenomenex). Online chromatography was performed using a Dionex Ultimate 3000 UHPLC (Thermo Fisher Scientific) at a flow rate of 300 nl/min. Peptides were separated and eluted into the mass spectrometer using a gradient of 3 to 35% acetonitrile (0.1% formic acid) over 65 min, followed by 5 min at 95% acetonitrile (0.1% formic acid). MS was performed using an Orbitrap Velos Pro (Thermo Fisher Scientific) operated in data-dependent mode. Survey scans (MS1) were performed using the Orbitrap over a scan range of 350 to 650 m/z and resolution setting of 60,000. A lock mass of 445.12003 m/z was used to achieve internal mass calibration. On the basis of MS1 scans, MS2 scans were performed using the ion trap, selecting the top ten most intense precursor (MS1) ions for fragmentation by collision-induced dissociation (CID) at 35% collision energy with a precursor isolation window of 2 m/z. MS2 scans were only collected on peptides with charge states of 2+ or 3+ with a minimum MS1 intensity of 50 counts. Advanced gain control (AGC) settings were 5 × 10^5^ for Orbitrap scans and 2 × 10^5^ for ion trap scans.

### Mass Spectrometry Analysis of TMT-labeled MHC-I Peptides

For TMT-labeled MHC-I peptides, lyophilized peptides were solubilized in 100 μl of 30% ACN in 50 mM HEPES (pH 8.5) and 10 μl of TMT reagents at a final concentration of 20 μg/ml in anhydrous ACN, mixed, and purified by SPE with 10 mg Oasis HLB cartridges (Waters). Lyophilized TMT-labeled peptides were resuspended in 6 μl of 1% formic acid and analyzed by LC-SPS-MS3. An aliquot of 2 μl was loaded onto a column and analyzed using an Orbitrap Fusion Lumos mass spectrometer (Thermo Fisher Scientific) coupled to a Proxeon EASY-nLC 1200 liquid chromatography (LC) pump (Thermo Fisher Scientific). Peptides were separated on a 100 μm inner diameter microcapillary column packed with 35 cm of Accucore C18 resin (2.6 μM, 150 Å, Thermo Fisher). Peptides were separated at a flow rate of ∼500 nl/min using a gradient of 3 to 22% acetonitrile (0.125% formic acid) over 120 min and analyzed by SPS-MS3. MS1 scans were acquired over an m/z range of 300 to 800, 60,000 resolution, AGC target of 5 × 10^5^, and maximum injection time of 250 ms. MS2 scans were acquired on MS1 ions of charge state 2+ to 3+ using an isolation window of 0.7 Th, CID activation with a collision energy of 35%, rapid scan rate, AGC target of 5000, and maximum injection time of 150 ms. MS3 scans were acquired using SPS of 15 isolation notches, m/z range of 100 to 1000, 15,000 resolution, AGC target of 5 × 10^5^, higher-energy collisional dissociation (HCD) activation at 55%, and maximum injection time of 300 ms. Each sample was injected twice with dynamic exclusion of 10 or 30 s (after 1 MS2, with an 8 ppm tolerance window). The global cycle time for the method was set at 3 s.

### MHC-I Peptide Identification and Analysis

MHC-I peptides were identified using a previously described targeted search strategy (42). Briefly, MHC-I peptides were predicted from all mouse proteins for the H2-D^b^ and H2-K^b^ alleles (FASTA database downloaded from UniProtKB December 2015) using NetMHC (43) with a rank cutoff of 2%, and these peptides were used to create a new FASTA database containing 56,479 entries. MS/MS data were searched using Sequest with “no cleavage” enzyme specificity with an MS1 tolerance of 5 ppm and MS2 tolerance of 0.5 Da. False discovery rates were controlled to 5% using Percolator. Searches were implemented in Protein Discoverer (PD) version 2.2 (Thermo Fisher Scientific). Label-free quantitation was also implemented in PD version 2.2 using the Minora peak alignment algorithm. All peptides were normalized based on the summed peptide intensity for the entire sample. For TMT-labeled MHC-I peptides, TMT was set as a fixed modification (229.162932) on lysine residues and peptide N-termini and carbamidomethylation (15.99492) as a fixed modification on cysteine. The summed reporter ion S/N for all spectral matches (PSMs) for each peptide was used for relative quantitation and normalized within each channel using the summed S/N for all compared peptides. Averages of the technical duplicates for each experimental group were used for analysis. Channel 131C for the B22.249 IP and channel 129C for the Y3 IP were removed for analysis due to poor TMT labeling.

### T Cell Activation Assay

For splenocytes containing CD8 T lymphocytes and antigen-presenting cells, spleens were harvested from mice and mechanically disrupted using the end of a syringe plunger. Cells were filtered through a 40 μm cell strainer and treated with ACK buffer (Thermo Fisher Scientific). The resulting single-cell suspension of splenocytes was cultured in RPMI 1640 containing 1% (vol/vol) Glutamax, 10% fetal bovine serum, 1× sodium pyruvate, 1× nonessential amino acids, and 1× Antibiotic-Antimycotic (all obtained from Invitrogen) for *ex vivo* stimulation. Splenocytes (1 × 10^6^ cells/well in a 96-well plate) were cultured in the presence of peptide (10 μg/ml) and purified anti-mouse CD28 antibody (1 μg/ml) for 24 h. The concentration of IFNγ in the supernatant was assessed using the IFNγ ELISA kit following the manufacturer’s instructions. For intracellular IFNγ staining for flow cytometry analysis, cells were treated with Brefeldin A at 18 h post peptide stimulation and incubated for an additional 6 h prior to Fc receptor blocking with anti-mouse CD16/32 for 25 min at 4 °C in FACS buffer. Cells were then stained for T cell markers (CD3, CD4, CD8) and then intracellular IFNγ using the Foxp3/transcription factor staining buffer set following the manufacturer’s instructions.

### Experimental Design and Statistical Rationale

For flow cytometry analysis of MHC/PDL-1 expression, reovirus infectivity, and oncolysis of MCA205 and ID8 cells *in vitro*, three technical replicates for each treatment group were performed in three independent experiments. For the quantification of PD-1 and PDL-1 expression on CD3 and CD8 T cells *in vivo*, five biological replicates were used. Quantitative PCR analysis was performed in three technical replicates for each treatment group in three independent experiments. For MHC-I peptide isolation, 2 to 5 tumor samples were pooled for each treatment group (for 1 g of sample each), and IP was performed in two technical replicates. T cell activation assay to test the immunogenicity of DEMHCPs was performed using splenocytes pooled from two biological replicates (PBS-treated or reovirus + ICB-treated each). Stimulation with individual peptides was performed without replicates due to the limited amount of synthetic peptides available. Control groups (splenocytes cultured with no peptide, splenocytes cultured with anti-mouse CD28 antibody only, splenocytes cultured with 2 μg/ml of Concanavalin A) were performed in three technical replicates.

Depending on the indicated experiment, one-way analysis of variance (ANOVA) with Bonferroni posttest or a two-tailed Student *t* test with 95% confidence interval was performed, and *p* values of <0.05 were considered significant. Statistical significance is represented by asterisks above the bar graphs (∗∗∗∗*p* ≤ 0.0001, ∗∗∗*p* ≤ 0.001, ∗∗*p* ≤ 0.01, ∗*p* ≤ 0.05, n.s. *p* > 0.05).

## Results

### Oncolytic Reovirus-induced Alteration of the Tumor MHC-I Peptidome Is Dictated by Cancer Type and Susceptibility to OV

**Fig. 1:**
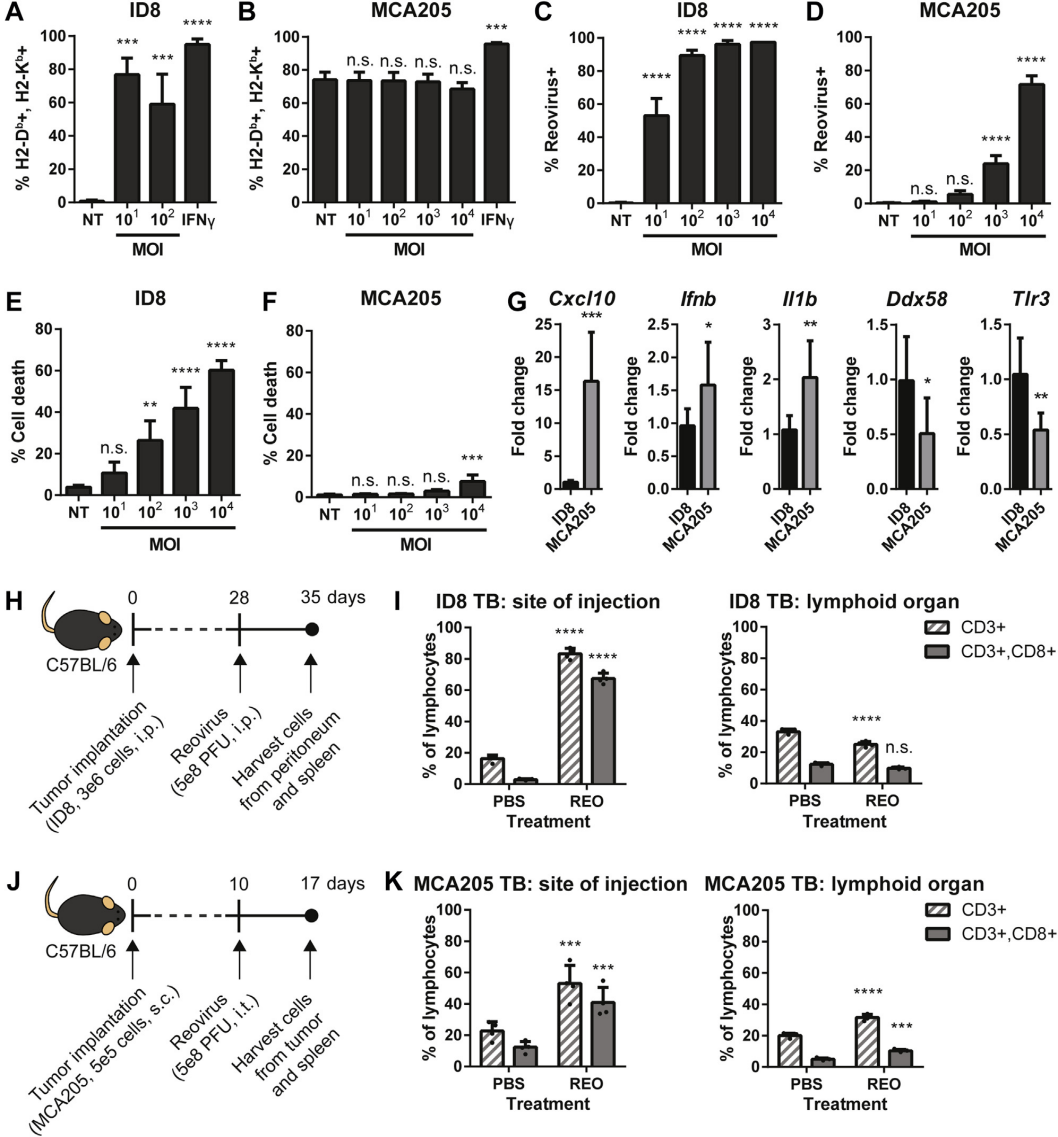
**Comparison of reovirus-modulated changes in ID8 and MCA205 models.** MHC-I H2-D^b^ and H2-K^b^ expression levels in (*A*) ID8 and (*B*) MCA205 cancer cells *in vitro*. Cancer cells were infected with reovirus at various multiplicity of infection (MOI; 10–10,000) for 24 h, and H2-D^b^+, H2-K^b^+ cells were quantified by flow cytometry. Stimulation with IFNγ (100 units/ml [U/ml]) was included as a positive control. Reovirus infectivity in (*C*) ID8 and (*D*) MCA205 cells *in vitro*. Cancer cells were infected at various MOIs for 24 h, and reovirus+ cells were quantified by flow cytometry. Reovirus-mediated oncolysis in (*E*) ID8 and (*F*) MCA205 cells *in vitro*. Cancer cells were infected at various MOIs for 24 h, and late apoptotic (Annexin V+, 7AAD+) cells were quantified by flow cytometry. *G*, quantitative PCR analysis of antiviral gene expression. Nontreated ID8 and MCA205 cells were collected for RNA extraction and cDNA synthesis to measure the expression of *Cxcl10*, *Ddx58*, *Ifnb*, *Il1b*, and *Tlr3* by qRT-PCR using gene-specific primers. All values were first normalized to *Gapdh* and compared with ID8 control. Reovirus-mediated tumor immune cell infiltration in (*H* and *I*) ID8 and (*J* and *K*) MCA205 models *in vivo*. CD3 and CD8 T cell levels were measured by flow cytometry in tumor-bearing (TB) mice at both the site of injection (tumor) and lymphoid organ (spleen). Statistical analysis was performed using a Student *t* test or one-way ANOVA coupled with a Bonferroni posttest. Data are representative of three independent experiments. *Asterisks* shown immediately on *top* of the bars signify the *p* values obtained by comparing the respective data against the control group (nontreated [NT], ID8 or PBS). n.s. *p* > 0.05, ∗*p* ≤ 0.05, ∗∗*p* ≤ 0.01, ∗∗∗*p* ≤ 0.001, ∗∗∗∗*p* ≤ 0.0001.

Based on our previous findings that reovirus induces the presentation of novel MHC-I peptides in the MOSE ID8 ovarian peritoneal carcinomatosis model (30), we investigated reovirus-induced changes in the tumor MHC-I peptidome of a solid tumor model of MCA205 fibrosarcoma. There are a few reasons why we chose the MCA205 model. Unlike ID8 cancer cells, which express low basal levels of MHC-I molecules that are upregulated in response to reovirus infection (Fig. 1*A*), MCA205 cancer cells express constitutively high levels of MHC-I molecules that remain unaffected by reovirus infection (Fig. 1*B*). We reasoned that MCA205 provides a good model to examine reovirus-mediated changes to the MHC-I peptidome without the need to account for changes to the MHC-I expression as a possible confounding variable. Moreover, MCA205 and ID8 models show differential susceptibility to reovirus infection *in vitro* (Fig. 1, *C* and *D*), with MCA205 cells being highly resistant to infection. Consequently, oncolysis, as measured by Annexin V+, 7AAD+ dead cells (Fig. 1, *E* and *F*), was reflective of infectivity levels where the reovirus-resistant MCA205 cells maintained cell viability at all multiplicity of infection (MOI) tested. Differences were also observed in mRNA levels of several markers that are known to be involved in antiviral responses (Fig. 1*G*). When nontreated ID8 and MCA205 cells were compared, higher basal levels of antiviral response genes (*e.g.*, *Cxcl10*, *Ifnb*, *Il1b*) and lower basal levels of dsRNA sensor genes (*e.g.*, *Ddx58*, *Tlr3*) were observed in MCA205 cells, which would limit viral infection/replication and detection, respectively. Contrasting response to reovirus infection in the two tumor models was also evident *in vivo*. While reovirus alters the TME and drives an increase in the levels of CD3 and CD8 tumor-infiltrating lymphocytes in both the ID8 and MCA205 models, it did so at a much higher level in the ID8 model (Fig. 1, *H* and *I*) as compared with the MCA205 model (Fig. 1, *J* and *K*). Thus, MCA205 fibrosarcoma cells additionally allow us to explore if reovirus exposure drives changes in the MHC-I peptidome in cancer cells that are relatively resistant to infection by this OV.

**Fig. 2:**
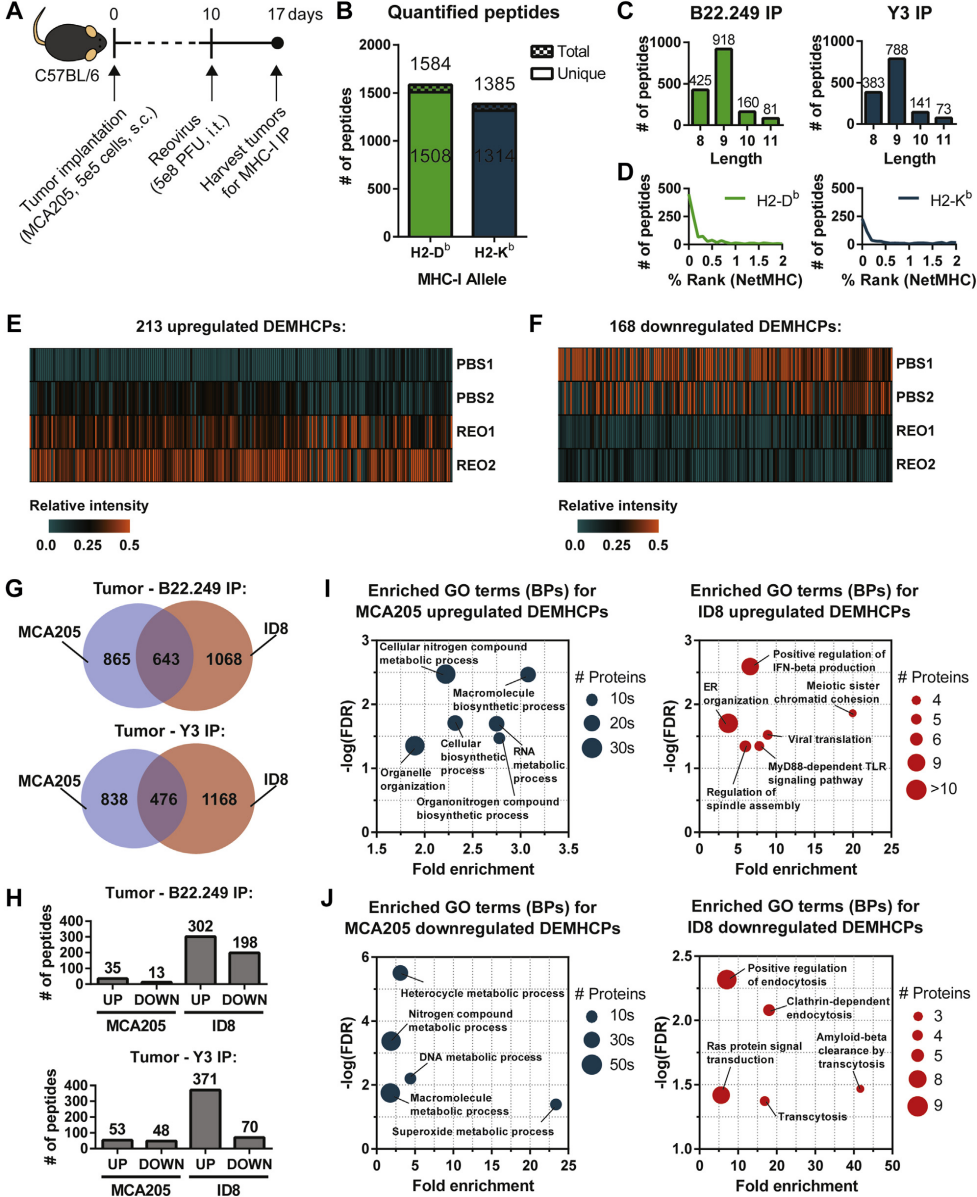
**Label-free quantitation of oncolytic reovirus-modulated MCA205 MHC-I peptidome.***A*, experimental setup for MHC-I peptidome analysis of MCA205 tumors following reovirus treatment. MCA205 tumor-bearing mice were injected with either PBS (n = 3, pooled) or reovirus (5 × 10^8^ PFU, i.t.; n = 5, pooled), and tumors were harvested for MHC-I peptidome and mass spectrometry analysis with label-free quantitation. *B*, number of total and unique H2-D^b^- and H2-K^b^-specific peptides quantified in the experiment. *C*, length distribution of the quantified MHC-I peptides. *D*, predicted binding affinity (NetMHC % rank) of the quantified MHC-I peptides. Peptides that are <0.5% rank are considered strong binders, whereas those that are 0.5 to 2% rank are weak binders. Relative intensities of H2-D^b^- and H2-K^b^-specific peptides that are specifically (*E*) upregulated (log_2_[reovirus/PBS] ≥ 1) or (*F*) downregulated (log_2_[reovirus/PBS] ≤ −1) by reovirus (REO) as compared with PBS-treated tumor-bearing control mice. *G*, number of distinct and overlapping H2-D^b^ (B22.249 IP) or H2-K^b^ (Y3 IP) peptides from the MCA205 and ID8 datasets. *H*, number of upregulated (UP) or downregulated (DOWN) MHC-I peptides observed for the MCA205 and ID8 models out of the overlapping peptides in common between the two datasets. *I*, enriched GO terms (biological process, BP) in the source proteins of upregulated DEMHCPs. *J*, enriched GO terms (biological process, BP) in the source proteins of downregulated DEMHCPs.

To test this, C57BL/6 mice were implanted with MCA205 cells, and the resultant tumors were administered with reovirus as shown in Figure 2*A*. Tumors from PBS-treated (control) or reovirus-treated MCA205 TB mice were collected and processed for MS-based MHC-I peptidome analysis. In this initial investigation, our label-free quantitation resulted in a dataset of 1508 unique H2-D^b^-specific peptides and 1314 unique H2-K^b^-specific peptides, totalling 2822 unique MHC-I peptides matching to 2290 protein accessions (Fig. 2*B*, supplemental Tables S1 and S2). The majority of the MHC-I peptides we identified displayed typical amino acid length distributions for MHC-I peptides (Fig. 2*C*) and had NetMHC-predicted binding affinities less than 0.5% rank (869 H2-D^b^ peptides were <0.5% [strong binders] and 224 were 0.5–2% [weak binders]; 436 H2-K^b^ peptides were <0.5% [strong binders] and 190 were 0.5–2% [weak binders]) (Fig. 2*D*), thus confirming the robustness of our MHC-I peptidome precipitation, analysis, and detection protocol. Of these, 213 were DEMHCPs that were upregulated (log_2_[reovirus/PBS] ≥ 1) in response to reovirus treatment, representing 7.5% of the total MHC-I peptides quantified in the experiment (Fig. 2*E*). Here, we also investigated the downregulated DEMHCPs (log_2_[reovirus/PBS] ≤ −1), which we hypothesized show reduced expression due to possible immunoediting by cognate CD8 T cells, and identified 168 downregulated DEMHCPs in response to reovirus treatment, representing 6.0% of the total MHC-I peptides (Fig. 2*F*). These data showed that oncolytic reovirus modulates the expression of the MHC-I peptidome of cancer cells that are relatively resistant to infection and oncolysis.

One noticeable difference between the present study’s MCA205 MHC-I peptidome dataset and our previously published ID8 MHC-I peptidome dataset (30) was the number of peptides induced by reovirus treatment. Similar numbers of peptides were quantified from MHC-IP of MCA205 and ID8 tumors, and we even identified peptides that are common between the two datasets (Fig. 2*G*). However, we observed that out of the 643 H2-D^b^ and 476 H2-K^b^ overlapping peptides, higher numbers of upregulated and downregulated DEMHCPs were identified for the ID8 model (Fig. 2*H*), suggesting that reovirus-modulated changes to the MHC-I peptidome are relatively less evident in the MCA205 model. In addition, gene ontology (GO) enrichment analysis using the PANTHER Classification System (44) of the source proteins of upregulated DEMHCPs revealed that MCA205 DEMHCPs were enriched in cellular biosynthetic and metabolic biological processes (BPs) while ID8 DEMHCPs were enriched in viral defense BPs (Fig. 2*I*). For the downregulated DEMHCPs, metabolism-related BPs were enriched in the MCA205 dataset while endocytosis-related BPs were enriched in the ID8 dataset (Fig. 2*J*). Overall, the comparison of the MHC-I peptidome datasets of MCA205 fibrosarcoma and ID8 ovarian cancer cells identified OV-induced oncolysis and tissue origin as possible dictators of therapy-induced cancer MHC-I peptidome changes in response to oncolytic reovirus treatment.

### Immune Checkpoint Blockade Further Augments the MHC-I Peptidome Changes Induced by Oncolytic Reovirus

**Fig. 3:**
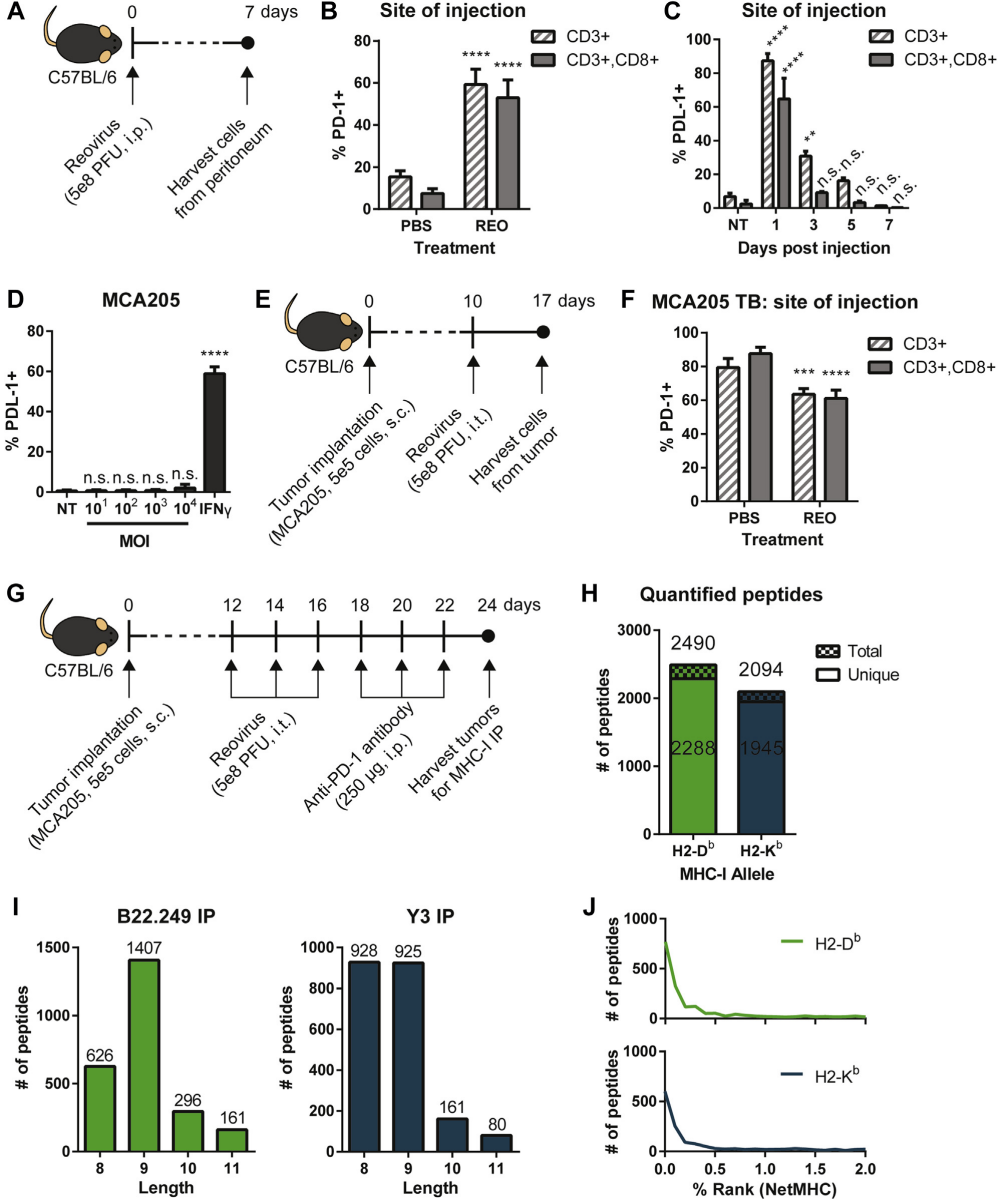
**TMT-based multiplexing platform analysis of reovirus and ICB combination-modulated MCA205 MHC-I peptidome.***A*, schematic of reovirus infection *in vivo* to analyze immune checkpoint expression in non-tumor-bearing mice. *B*, flow cytometry analysis of PD-1 expression on CD3 and CD3+CD8 T cells from the site of injection (peritoneum) of C57BL/6 mice (n = 5) at 7 days post injection, compared with PBS-treated control mice. A two-tailed Student *t* test with 95% confidence interval was performed. *C*, PDL-1 expression on CD3 and CD3+CD8 T cells from the site of injection (peritoneum) at 1, 3, 5, 7 days post injection were also quantified by flow cytometry (n = 2 at each timepoint). *D*, PDL-1 expression level on MCA205 cancer cells *in vitro*. Cells were infected with reovirus at various MOIs for 24 h, and PDL-1+ cells were quantified by flow cytometry. Stimulation with IFNγ (100 U/ml) was included as a positive control. Data are representative of three independent experiments. One-way ANOVA coupled with a Bonferroni posttest was performed. *E*, schematic of intratumoral reovirus administration *in vivo* to analyze immune checkpoint expression in MCA205 tumor-bearing (TB) mice. *F*, flow cytometry analysis of PD-1 expression on CD3 and CD3+CD8 T cells from the site of injection (tumor) of C57BL/6 mice (n = 4) at 7 days post injection, compared with PBS-treated control mice. *G*, experimental setup for MHC-I peptidome analysis of MCA205 tumors following reovirus and ICB combination treatment (n = 2–5 per treatment group, pooled). Immunoaffinity-purified MHC-I peptides were analyzed by TMT-based multiplexed quantitation. *H*, number of total and unique H2-D^b^- and H2-K^b^-specific peptides quantified in the experiment. *I*, length distribution of the quantified MHC-I peptides. *J*, predicted binding affinity (NetMHC % rank) of the quantified MHC-I peptides. *Asterisks* shown immediately on *top* of the bars signify the *p* values obtained by comparing the respective data against the nontreated (PBS or NT) control group. n.s. *p* > 0.05, ∗*p* ≤ 0.05, ∗∗*p* ≤ 0.01, ∗∗∗*p* ≤ 0.001, ∗∗∗∗*p* ≤ 0.0001.

Given the minimal changes to the MHC-I peptidome observed in the MCA205 tumors following reovirus treatment, we sought to improve this by adding ICB therapy. We reasoned that ICB is an ideal candidate for combination treatment with reovirus since virus infection induces an upregulation of immune checkpoint ligand/receptor expression on cancer cells and immune cells (45, 46). Here, we chose to perform the blocking of programmed cell death protein 1 (PD-1), the expression of which is upregulated on CD3 and CD8 T cells at the site of injection (*i.e.*, peritoneum) in response to reovirus injection (Fig. 3, *A* and *B*). Of note, programmed death-ligand 1 (PDL-1), on the other hand, is not upregulated on CD3 and CD8 T cells at the site of infection, despite an initial peak at 1 day post injection, (Fig. 3*C*) nor on MCA205 cancer cells in response to reovirus infection *in vitro* (Fig. 3*D*). In the context of a tumor microenvironment (Fig. 3*E*), tumor-infiltrating CD3 and CD8 T cells constitutively expressed high levels of PD-1, which decreased following intratumoral reovirus administration (Fig. 3*F*). Nevertheless, PD-1 expression levels were still high and rendered this tumor model susceptible to ICB therapy, and thus PD-1 rather than PDL-1 was our target of choice for ICB. We hypothesized that ICB-mediated activation of otherwise suppressed T cells would result in cancer immunoediting with subsequent alteration of the MHC-I peptidome repertoire. To examine this, MCA205 TB mice were treated with reovirus and anti-PD-1 antibody as per the schematic shown in Figure 3*G*, and then tumors were harvested for MS-based MHC-I analysis. In this experiment, we utilized a TMT-based platform for a multiplexed quantitative analysis of MHC-I peptides (31). We observed 2288 unique H2-D^b^-specific and 1945 unique H2-K^b^-specific peptides, matching to 1308 protein accessions (Fig. 3*H*, supplemental Tables S3 and S4). The amino acid length distribution (Fig. 3*I*) and NetMHC-predicted binding affinities (Fig. 3*J*) once again support our dataset as *bona fide* MHC-I peptides.

**Fig. 4:**
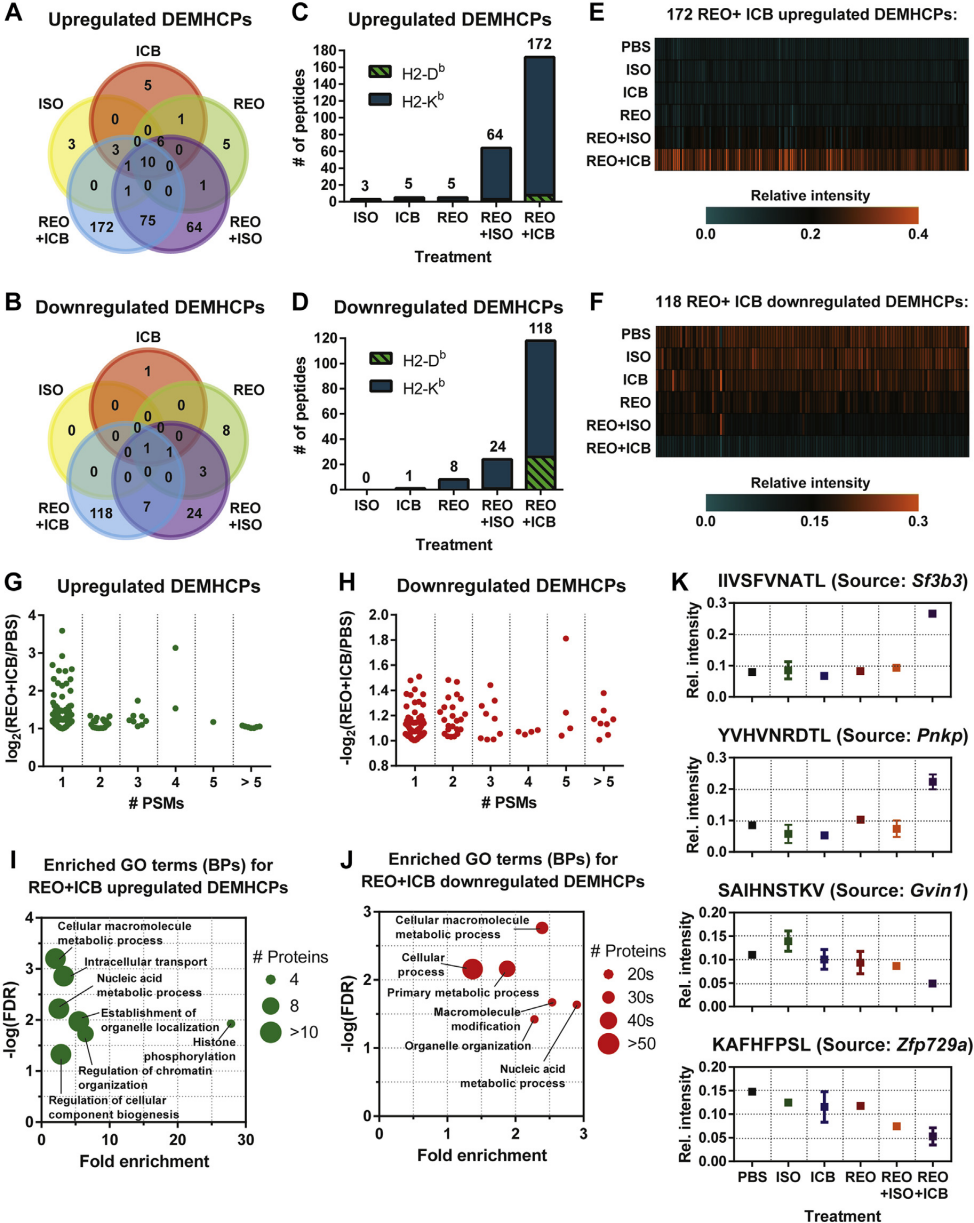
**Characterization of upregulated and downregulated DEMHCPs of reovirus and ICB combination therapy.** Number of unique and overlapping (*A*) upregulated (log_2_[fold change] ≥ 1) or (*B*) downregulated (log_2_[fold change] ≤ −1) H2-D^b^- and H2-K^b^-specific DEMHCPs observed in the different treatment groups as compared to PBS control. Numbers of unique (*C*) upregulated or (*D*) downregulated DEMHCPs from each treatment group as compared to PBS control. Relative intensities of MHC-I peptides that are specifically (*E*) upregulated or (*F*) downregulated by reovirus and ICB combination therapy. Number of PSMs and fold change levels of the combination therapy (*G*) upregulated and (*H*) downregulated DEMHCPs. Enriched GO terms (biological process) in the source proteins of combination therapy (*I*) upregulated and (*J*) downregulated DEMHCPs. *K*, representative examples of MHC-I peptides that are upregulated or downregulated by combination therapy. ISO, isotype control antibody; ICB, immune checkpoint blockade (anti-mouse PD-1 antibody); REO, reovirus.

Next, we compared each treatment group to the PBS-treated control group to identify MHC-I peptides that are upregulated (log_2_[fold change] ≥ 1; Fig. 4*A*) or downregulated (log_2_[fold change] ≤ −1; Fig. 4*B*). While there were some overlapping DEMHCPs in common between treatments, especially when comparing reovirus and isotype control antibody or anti-PD-1 antibody combination therapy (henceforth referred to as REO + ISO or REO + ICB, respectively), we focused on DEMHCPs that are unique to each treatment considering our interest in cancer immunoediting (Fig. 4, *C* and *D*). We observed the highest number of DEMHCPs due to REO + ICB combination therapy, with 172 upregulated (Fig. 4*E*) and 118 downregulated (Fig. 4*F*), representing 4.1% and 2.8% of the total MHC-I peptides identified, respectively. These REO + ICB DEMHCPs exhibited a range of fold change levels and number of peptide spectrum matches (PSMs) (Fig. 4, *G* and *H*). GO enrichment analysis of the source proteins of the REO + ICB DEMHCPs revealed enriched BPs such as nucleic acid metabolic process, macromolecular modification, and organelle organization (Fig. 4, *I* and *J*). These are exemplified by MHC-I peptides from *Sf3b3*, *Pnkp*, *Gvin1*, and *Zfp729a* (Fig. 4*K*). Altogether, these data show the unique changes to the MCA205 MHC-I peptidome with the identification of DEMHCPs specific to reovirus and ICB combination treatment. Most importantly, these results demonstrate that the use of ICB within a combinatorial treatment can augment therapy-induced MHC-I peptidome changes during oncolytic virus-based cancer therapies.

### Differentially Expressed MHC-I Peptides Observed Following Reovirus + ICB Combination Therapy Contain Biologically Active Antigenic Antitumor CD8 T Cell Epitopes

**Fig. 5:**
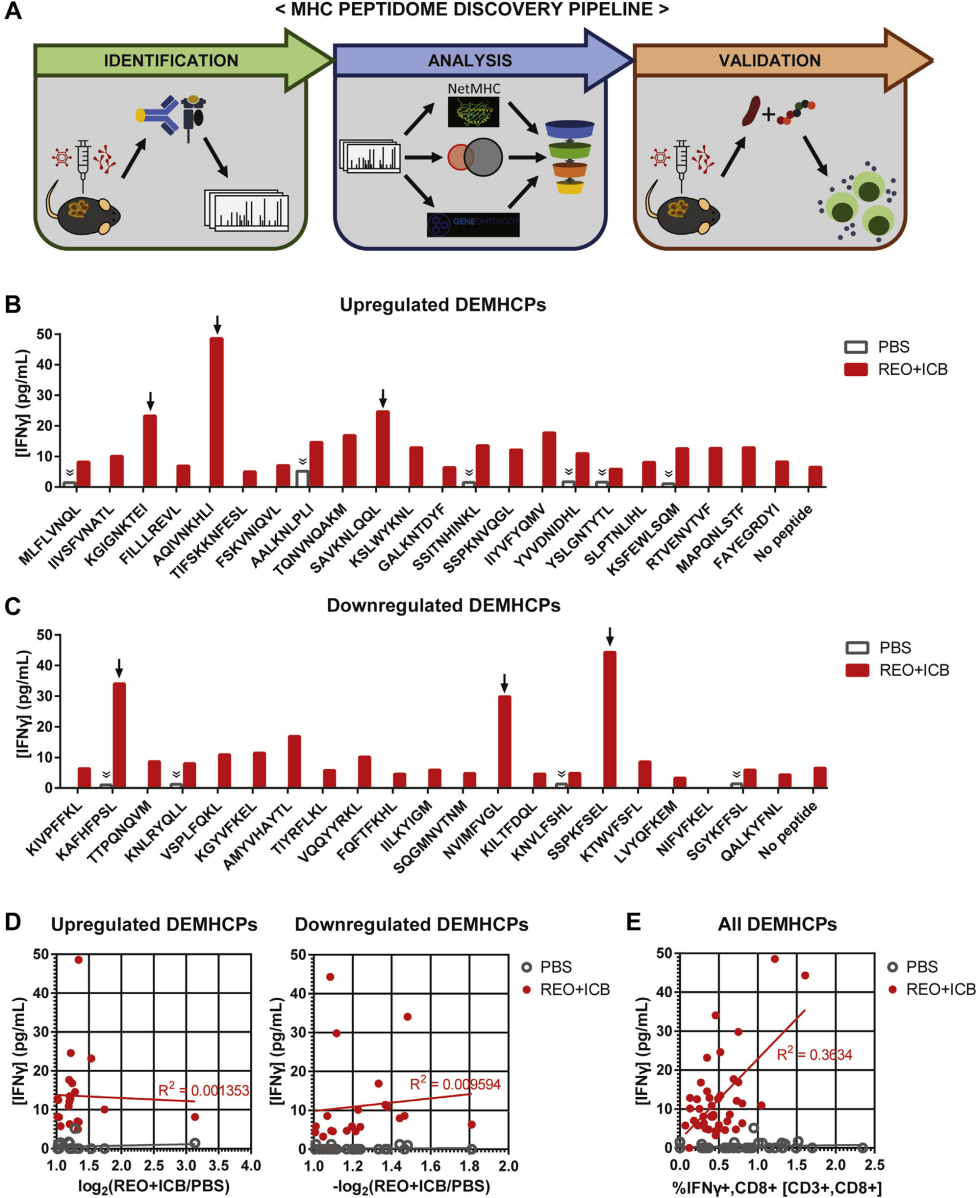
**Functional validation of reovirus and ICB combination therapy DEMHCPs as antitumor CD8 T cell epitopes.***A*, schematic diagram of the MHC-I peptidome discovery pipeline, from MHC-I peptide identification, analysis, to biological validation. Concentration of secreted interferon-gamma (IFNγ) as measured by ELISA following *ex vivo* stimulation of splenocytes with (*B*) upregulated DEMHCPs or (*C*) downregulated DEMHCPs. Splenocytes from PBS- or reovirus and ICB (REO + ICB)-treated MCA205 tumor-bearing (TB) mice were cultured with the peptides for 24 h, and then supernatants were collected for ELISA analysis. *Arrows* and *chevron-double-down* symbols indicate peptides that induced IFNγ levels greater than the cutoff value (mean + 3 × standard deviation) of the negative controls (unstimulated splenocytes; 0 pg/ml for PBS and 17.77 pg/ml for REO + ICB). *D*, level of secreted IFNγ measured by ELISA and the corresponding fold change level measured by MHC-I analysis for each validated peptide. *Red* and *gray lines* show the best fit linear regression. *E*, level of secreted IFNγ measured by ELISA and the corresponding intracellular IFNγ level measured by flow cytometry for each validated peptide. *Red* and *gray lines* show the best fit linear regression.

Not all MHC-I peptides present in a cell are antigenic and are rather often involved in homeostatic immunoregulation. Thus, to realize the role of MHC-I peptides as an antigenic epitope for CD8 T cell recognition, their capacity to stimulate antigen-specific CD8 T cells must be tested (Fig. 5*A*). Hence, we next investigated the biological activity of the reovirus and ICB combination therapy-modulated DEMHCPs. We chose 22 upregulated and 21 downregulated DEMHCPs with number of PSMs greater than or equal to 2 (Table 1). These 43 peptides were synthesized, and their capacity to elicit CD8 T cell stimulation in splenocytes of untreated (PBS control) or REO+ICB-treated TB mice was measured by an interferon-gamma (IFNγ) ELISA validation screen. Out of the 22 upregulated DEMHCPs, three peptides produced a strong IFNγ response (greater than the cut-off of [mean + 3 × standard deviation] of the negative controls) in splenocytes of REO+ICB-treated TB mice (Fig. 5*B*, arrows). Interestingly, three out of the 21 downregulated DEMHCPs also induced a strong response in splenocytes of REO+ICB-treated TB mice (Fig. 5*C*, arrows). We also found a few peptides that elicited a positive IFNγ response in splenocytes of PBS-treated control TB mice; although above the cutoff values, these responses were low (Fig. 5, *B* and *C*, chevron-double-down symbol). Furthermore, there was no correlation between the MHC-I peptide abundance fold change levels and IFNγ response (Fig. 5*D*). If anything, the immunogenic peptides tend to have lower fold change levels. We also confirmed the immunogenicity of the peptides by staining the stimulated splenocytes for intracellular IFNγ to be analyzed by flow cytometry. Comparison of the ELISA and flow cytometry data from splenocytes of REO + ICB-treated TB mice showed a slight trend of positive correlation of immunogenicity (Fig. 5*E*). Due to the low percentage of IFNγ+, antigen-specific CD8 T cells detected by flow cytometry, ELISA may be a more reliable measure of an immunogenicity screen. Nevertheless, these data strongly support that reovirus+ICB therapy-induced DEMHCPs can activate cognate CD8 T cells. As CD8 T cells are the main mediators of OV+ICB therapeutic effects, these therapy-induced DEMHCPs are of importance in the context of antitumor immunity and therapeutic efficacy.

**Table 1:** List of reovirus and ICB combination therapy DEMHCPs for immunogenicity validation

Sequence	Source protein gene	MHC allele	# PSMs	log_2_(REO + ICB/PBS)
MLFLVNQL	*Pcid2*	Kb	4	3.14
IIVSFVNATL	*Sf3b3*	Db	3	1.74
KGIGNKTEI	*Nsd3*	Kb	4	1.54
FILLLREVL	*Marchf6*	Kb	3	1.34
AQIVNKHLI	*Eif2b3*	Kb	2	1.34
TIFSKKNFESL	*Rpn2*	Kb	2	1.33
FSKVNIQVL	*Oasl2*	Kb	3	1.32
AALKNLPLI	*Acsl3*	Kb	2	1.29
TQNVNQAKM	*Maged1*	Kb	2	1.24
SAVKNLQQL	*Nprl3*	Kb	3	1.22
KSLWYKNL	*Il17rc*	Db	2	1.22
GALKNTDYF	*Nt5c3a*	Kb	2	1.20
SSITNHINKL	*Sema5a*	Kb	3	1.20
SSPKNVQGL	*Siglec1*	Kb	2	1.20
IIYVFYQMV	*Kifap3*	Db	2	1.20
YVVDNIDHL	*Cop1*	Kb	3	1.19
YSLGNTYTL	*Gpsm1*	Kb	3	1.06
SLPTNLIHL	*Ubr2*	Kb	7	1.04
KSFEWLSQM	*Dync1h1*	Db	2	1.03
RTVENVTVF	*Vat1*	Kb	2	1.02
MAPQNLSTF	*Dnajb11*	Kb	2	1.02
FAYEGRDYI	*H2-Q6*	Kb	20	1.02
KIVPFFKL	*Dync1h1*	Kb	5	−1.81
KAFHFPSL	*Zfp729a*	Kb	2	−1.48
TTPQNQVDM	*Chmp1b1*	Db	2	−1.47
KNLRYQLL	*Matr3*	Kb	3	−1.44
VSPLFQKL	*Mars1*	Kb	6	−1.38
KGYVFKEL	*Tlr7*	Kb	2	−1.37
AMYVHAYTL	*Psma3*	Db	2	−1.33
TIYRFLKL	*Fem1c*	Kb	7	−1.24
VQQYYRKL	*Prex1*	Kb	2	−1.23
FQFTFKHL	*Dnaja2*	Kb	3	−1.21
IILKYIGM	*Arl6ip1*	Kb	2	−1.20
SQGMNVTNM	*Ep300*	Db	6	−1.17
NVIMFVGL	*Srp54c*	Kb	2	−1.12
KILTFDQL	*Rpl18*	Kb	5	−1.10
KNVLFSHL	*Prkar1b*	Kb	2	−1.09
SSPKFSEL	*Wdr45*	Kb	4	−1.08
KTWVFSFL	*Cilp*	Kb	4	−1.07
LVYQFKEM	*Elf4*	Kb	4	−1.05
NIFVFKEL	*Rapgef6*	Kb	2	−1.03
SGYKFFSL	*Wipi2*	Kb	3	−1.01
QALKYFNL	*Sel1l*	Kb	6	−1.01

Abbreviations: ICB, immune checkpoint blockade; MHC, major histocompatibility complex; PSM, peptide spectrum match; REO, reovirus.

## Discussion

Here, we present the first report on the oncolytic reovirus + ICB combination therapy-induced changes to the tumor MHC-I peptidome. Using a relatively OV-resistant cancer model, we demonstrate that despite the low susceptibility of cancer cells, oncolytic reovirus changes the MHC-I peptidome of MCA205 cancer cells, albeit at a lower magnitude than that in OV-susceptible ID8 ovarian cancer cells. Next, we found that the reovirus-induced modulation of MHC-I peptidome in OV-resistant cells can be further augmented *via* an addition of ICB agents within the OV therapeutic regimen. From the clinical perspective and in line with our previous reports on therapy-induced changes to the MHC-I peptidome repertoire (30, 31), here we show that the combination therapy-induced DEMHCPs have therapeutic potential in activating cognate antitumor CD8 T cells. These analyses provide an insight on how the tumor MHC-I peptidome changes in response to cancer immunotherapies and highlight immunological nuances that could be harnessed to overcome the adaptive therapy resistance of cancers.

In this study, we initially evaluated reovirus-induced changes to the MHC-I peptidome of a solid tumor model MCA205 fibrosarcoma and observed a low number of upregulated DEMHCPs, as compared to our previously published dataset on the MOSE ID8 ovarian cancer model. Further investigation revealed that this disparity was not only quantitative but also qualitative and possibly originated due to differential OV susceptibility, basal antigen presentation capacity, “hot” or “cold” nature, and tissue of origin within the cancers studied. Thus, our findings highlight the importance of context-dependent considerations for MHC-I peptidome analyses where certain tumors may be more suitable for MHC-I peptidome characterization. This is especially important when examining therapy-induced changes to the MHC-I peptidome landscape. One should assess the status of the antigen processing and presentation pathway of the tumors and determine whether the particular therapy under consideration is able to restore (or at least influence) the antigen presentation pathway (47). Baseline expression level of MHC-I molecules may also contribute to the overall quantitative changes observed in the MHC-I peptidome. However, even with the low number of upregulated DEMHCPs identified, the MCA205 model nevertheless showed that reovirus treatment induced changes to the tumor MHC-I peptide repertoire and provided a rationale for including ICB therapy in hopes of increasing the number of DEMHCPs.

We also employed a newly optimized TMT-based multiplexing platform for MHC-I peptidome analysis previously developed by our group (31). Multiplexing not only allows a comparison of 11 samples in a single experiment but also provides an accurate relative quantitation of low-abundance peptides (48). However, due to the constraints of the immuno-affinity purification-based method (*e.g.*, high amount of starting material required, which is contingent on animal number and tumor size), our experimental design lacked biological replicates. Nevertheless, in our proof-of-concept study, this high-throughput MHC-I peptidome discovery approach resulted in the identification of the antigenic targets for the combination therapy-modulated antitumor immunity. The resulting dataset of high-affinity MHC-I peptides (most of which have NetMHC % rank between 0.5 and 2), as expected for an immuno-affinity purification-based method of MHC-I peptides from whole cell lysates, is likely to favor the presentation of immunogenic MHC-I peptides. Contrary to what we expected, the addition of ICB to reovirus therapy did not significantly increase the number of DEMHCPs overall; that is, we observed less than 200 upregulated or downregulated DEMHCPs, which is a relatively low number. In any case, the combination therapy resulted in the highest number of DEMHCPs, especially compared with that of reovirus monotherapy, and emphasized the potential therapeutic advantage of the reovirus and ICB combination therapy.

Moreover, we also observed a high number of DEMHCPs due to reovirus and isotype control antibody combination treatment, providing further evidence to support the role of Fc gamma receptors (FcγR) in the activities of immunomodulatory antibodies as reviewed by Stewart and colleagues (49). Previous studies have shown the FcγR-dependent activity of anti-mouse CTLA-4 antibodies in mouse tumor models (50, 51, 52). Thus, the change in the MHC-I peptidome we observed due to the reovirus and isotype control antibody treatment may be due to nonspecific binding of the isotype control antibody to FcγR. This effect, as well as the one observed due to REO + ICB combination therapy, was observed only in the presence of reovirus since the proinflammatory stimuli were necessary to drive the expression of FcγR-expressing, or PD-1-expressing, effector cells in the tumor. These results support the role for OVs as primers ahead of the administration of ICB treatment (7) and provide an additional rationale for the use of OV+ICB combinations for enhanced anticancer therapeutic efficacy.

Since most MHC-I peptides in cells carry an immune homeostatic function and thus are not immunogenic, we added an additional step in the MHC-I peptidome discovery pipeline and validated the immunogenicity of the DEMHCPs found in our study. In the context of cancer immunotherapies, wherein functionally active antitumor CD8 T cells act as the main mediators of therapeutic effects, discovery of biologically active MHC-I peptides is highly clinically relevant. Out of the 43 peptides tested in the T cell activation-based validation screen, we observed six that stimulated high IFNγ responses in cognate CD8 T cells. As expected, many MHC-I peptides identified by the immuno-affinity purification and MS analysis approach failed to produce IFNγ responses, underlining the value in assessing antigen-specific T cell activity. We also noted that most of the 43 DEMHCPs were H2-K^b^-restricted, despite the fact that similar numbers of H2-D^b^ and H2-K^b^ peptides were identified overall, and allele specificity was not taken into consideration when selecting these DEMHCPs. It has previously been shown that H2-K^b^ is exported more rapidly to the cell surface than H2-D^b^ (53), so this faster turnover rate may have contributed to more H2-K^b^-bound upregulated/downregulated MHC-I peptides being observed. In addition, our observation of the lack of correlation between immunogenicity and fold change level as measured by MS-based MHC-I peptidome analysis suggests that other parameters aside from MS intensity fold change should be considered for selecting MHC-I peptides to assess immunogenicity. As such, stringent filters should be carefully applied in the workflow, which focuses on the identification of MHC-I peptides that are biologically active. A wide range of peptides should be represented, not only the highly upregulated or downregulated peptides, as there are other factors involved in determining immunogenicity besides MHC-I peptide abundance. Furthermore, our current data emphasize the need to consider the contribution of the downregulated DEMHCPs to the antitumor immune response, unlike our previous studies that had investigated only upregulated DEMHCPs. As we hypothesized, one mechanism through which these DEMHCPs are likely downregulated can be due to selective destruction, by cognate CD8 T cells, of tumor cells that express it. Thus, biologically active MHC-I peptides within downregulated DEMHCPs may potentially contribute toward cancer immunoediting and contain the target candidates for peptide vaccines. To firmly establish the role of downregulated DEMHCPs in immunoediting, further investigation is warranted. For instance, a temporal analysis of the MHC-I peptidome at different timepoints post treatment could provide an invaluable insight on the relationship between immunoediting and the MHC-I peptidome, especially with a therapeutic regimen showing tumor regression. Furthermore, vaccination experiments with the immunogenic DEMHCPs can be conducted to further boost the antitumor immune responses. The inclusion of nondifferentially expressed immunogenic MHC-I peptides can also expand the repertoire of antitumor T cell targets, but exome sequencing analysis will be required to ensure that such peptides are tumor-specific neoantigens. Ultimately, biologically active peptides found within upregulated and downregulated DEMHCPs hold implications for therapy-induced antitumor CD8 T cell responses.

In conclusion, this study further supports biological and therapeutic implications for therapy-induced changes to the MHC-I peptidome following combinatorial treatment with two emerging immunotherapies—OV and ICB. The elucidation of such therapy-driven DEMHCPs provides an insight on the alterations to the TME in response to therapy as well as identifies immunogenic peptides that can be exploited for the development of next-generation cancer immunotherapies.

## Data Availability

The mass spectrometry proteomics data have been deposited to the ProteomeXchange Consortium *via* the PRIDE partner repository with the dataset identifier PXD024369 and 10.6019/PXD024369.

## Supplemental data

This article contains supplemental data.

## Conflict of interest

The authors declare no competing interests.
